# Supplementary material for: Evaluating novel engagement mechanisms, yields and acceptability of tuberculosis screening at retail pharmacies in Ho Chi Minh City, Viet Nam
Source: PLOS Glob Public Health. 2022 Oct 17;2(10):e0000257. doi: 10.1371/journal.pgph.0000257 (PMC10021543; doi:10.1371/journal.pgph.0000257)
Supplement: S1 Table — (DOCX) [file pgph.0000257.s001.docx]

**Table S1**: Acceptability construct definitions

| **Construct** | **Definition** |
| --- | --- |
| Ethicality | The extent to which the intervention has a good fit with an individual’s value system |
| Intervention Coherence | The extent to which the participant understands the intervention and how it works |
| Burden | The perceived amount of effort that is required to participate in the intervention |
| Opportunity Cost | The extent to which benefits, profits or values must be given up to engage in the  intervention |
| Perceived Effectiveness | The extent to which the intervention is perceived as likely to achieve its purpose |
| Self-Efficacy | The participant’s confidence that they can perform the behavior(s) required to participate in the  intervention |
| Affective Attitude | How an individual feels about the intervention |
