# Supplementary material for: Evaluating novel engagement mechanisms, yields and acceptability of tuberculosis screening at retail pharmacies in Ho Chi Minh City, Viet Nam
Source: PLOS Glob Public Health. 2022 Oct 17;2(10):e0000257. doi: 10.1371/journal.pgph.0000257 (PMC10021543; doi:10.1371/journal.pgph.0000257)
Supplement: S2 Table — (DOCX) [file pgph.0000257.s002.docx]

**Table S2**: Cronbach's alpha (reliability coefficient)

|  | **N** | **Item-test correlation** | **Item-rest correlation** | **Cronbach's alpha** |
| --- | --- | --- | --- | --- |
| **Ethicality** |  |  |  |  |
| It is appropriate for a pharmacist to verbally screen | 100 | 0.57 | 0.49 | 0.83 |
| It is appropriate for a pharmacist to refer for X-ray without a physician consultation | 100 | 0.48 | 0.38 | 0.83 |
| **Intervention Coherence** |  |  |  |  |
| Screening can help customer get an early diagnosis of TB | 100 | 0.62 | 0.55 | 0.82 |
| Screening can ensure customers with TB receive quality-assured diagnostic care | 96 | 0.57 | 0.49 | 0.83 |
| **Opportunity Cost** |  |  |  |  |
| Screening takes away too much time from pharmacist's other activities | 100 | 0.32 | 0.17 | 0.85 |
| Screening would cost pharmacist money | 100 | 0.21 | 0.17 | 0.84 |
| Screening would cause pharmacist’s customers to not return | 98 | 0.54 | 0.43 | 0.83 |
| **Perceived Effectiveness** |  |  |  |  |
| Screening will help identify more people with TB | 100 | 0.71 | 0.65 | 0.82 |
| Screening will increase customer trust in pharmacist | 99 | 0.66 | 0.58 | 0.82 |
| **Affective Attitude** |  |  |  |  |
| Screening would be beneficial to pharmacy business | 100 | 0.73 | 0.66 | 0.81 |
| Screening would differentiate the service provided by pharmacist from others | 100 | 0.68 | 0.59 | 0.82 |
| **Self-Efficacy** |  |  |  |  |
| Confidence in ability to screen for TB symptoms using ACIS/SwipeRx app | 89 | 0.63 | 0.54 | 0.82 |
| Confidence in ability to refer eligible people for chest X-ray | 97 | 0.62 | 0.55 | 0.82 |
| **Burden*** |  |  |  |  |
| Time requirement for beneficiaries to be screened | 99 | 0.53 | 0.42 | 0.83 |
| Time requirement for beneficiaries to get a chest X-ray and be evaluated for TB | 97 | 0.39 | 0.28 | 0.84 |
| **Test scale** |  |  |  | **0.84** |
