# Supplementary material for: Evaluating novel engagement mechanisms, yields and acceptability of tuberculosis screening at retail pharmacies in Ho Chi Minh City, Viet Nam
Source: PLOS Glob Public Health. 2022 Oct 17;2(10):e0000257. doi: 10.1371/journal.pgph.0000257 (PMC10021543; doi:10.1371/journal.pgph.0000257)
Supplement: S3 Table — (DOCX) [file pgph.0000257.s003.docx]

**Table S3**: Comparison of training and implementation constructs among private pharmacists

|  | **n** | **Pharmacists making**  **≥1 CXR referral (n=50)** | | **Pharmacists making no CXR referrals (n=50)** | | **aOR (95%CI)** | **p-value** | **aOR (95%CI)** | **p-value** |
| --- | --- | --- | --- | --- | --- | --- | --- | --- | --- |
|  |  | **Mean**  **(95% CI)** | **Median (IQR)** | **Mean**  **(95% CI)** | **Median (IQR)** |  |  |  |  |
| Adequate training provided to use ACIS/SwipeRx app | 100 | 3.9 (3.7-4.1) | 4 (3-4) | 4.1 (3.9-4.2) | 4 (4-4) | 0.7 (0.4-1.3) | 0.209 | 0.6 (0.3-1.2) | 0.172 |
| **Implementation/Usability** |  |  |  |  |  |  |  |  |  |
| Concerns about customer confidentiality | 98 | 2.3 (1.9-2.6) | 2 (1-3) | 2.5 (2.1-2.8) | 2 (2-3) | 0.9 (0.6-1.2) | 0.456 | 0.9 (0.6-1.3) | 0.584 |
| Incentives motivated participation | 97 | 2.2 (1.9-2.5) | 2 (1-3) | 2.3 (2.0-2.6) | 2 (2-3) | 0.9 (0.6-1.3) | 0.633 | 1.0 (0.6-1.5) | 0.918 |
| Incentives fairly compensated for effort required to participate | 95 | 3.2 (3.0-3.4) | 3 (3-3) | 3.1 (2.9-3.4) | 3 (3-4) | 1.2 (0.7-2.1) | 0.444 | 1.3 (0.7-2.4) | 0.374 |
