# Supplementary material for: Evaluating novel engagement mechanisms, yields and acceptability of tuberculosis screening at retail pharmacies in Ho Chi Minh City, Viet Nam
Source: PLOS Glob Public Health. 2022 Oct 17;2(10):e0000257. doi: 10.1371/journal.pgph.0000257 (PMC10021543; doi:10.1371/journal.pgph.0000257)
Supplement: S1 Survey — (DOCX) [file pgph.0000257.s006.docx]

| **Before survey** |  |
| --- | --- |
| **ACIS ID***:* **____________________** | **SwipeRx ID: ______________________** |
| **Has the pharmacist referred yet?** [ ] Yes [ ] No |  |
| **Questions to be asked** |  |
| **1. Date of survey***:* *__ __/ __ __/__ __ __ __* | **2. Name of interviewer:** *__________________* |
| **3. Year of birth:** *__ __ __ __* | **4. Name of pharmacist:**  *__________________* |
| **5. Number of years working in the private pharmacy***: __________years* | |
| **6. During which year did you graduate from the pharmacy school?***: __ __ __ __* | |

**Instructions**: Please read these statements to pharmacists and ask them to pick the answer within the scale ranging from Strongly Agree to Strongly Disagree. In the statements, “I” means the pharmacist.

| **Questions** | **Strongly Agree** | **Agree** | **Neutral** | **Disagree** | | **Strongly Disagree** | **Refuse/**  **Unknown** |
| --- | --- | --- | --- | --- | --- | --- | --- |
| **7. The training** I received from the project officer **was adequate** to facilitate my use of the ACIS form in the SwipeRx app. |  |  |  |  | |  |  |
| 8. If a customer shows signs and symptoms of TB, it is **appropriate** for a pharmacist to verbally screen them for TB. |  |  |  |  | |  |  |
| 9. If a customer shows signs and symptoms of TB, it is **appropriate** for a pharmacist to refer this patient for **diagnostic testing** without a physician’s consultation. |  |  |  |  | |  |  |
| 10. By using the ACIS form in the SwipeRx app for screening for TB symptoms and referring for chest X-ray, I can help my customer get an **early diagnosis of TB**. |  |  |  |  | |  |  |
| 11. By using the ACIS form in the SwipeRx app for screening for TB symptoms and referring for chest X-ray, I ensure my customers with TB receive **quality-assured diagnostic care.** |  |  |  |  | |  |  |
| 12. Screening for TB symptoms and referring eligible people for chest X-ray would take away too much **time** **from my other activities**. |  |  |  |  | |  |  |
| 12a. If the answer= Agree or Strongly Agree=> If the data collection forms were shorter, would you still feel that screening took too much time away from other activities? | **[ ] Yes** | | **[ ] No** | | **[ ] Refuse/Unknown** | | |
| 13. Screening for TB symptoms and referring eligible people for chest X-ray **would cost me** **money**, in the form of either direct costs or lost opportunity costs. |  | |  | |  | | |
|  |  | |  | |  | | |
| **Questions** | **Strongly Agree** | **Agree** | **Neutral** | **Disagree** | | **Strongly Disagree** | **Refuse/**  **Unknown** |
| 14. I am concerned that screening for TB symptoms and referring eligible people for chest X-ray would **cause my customers to not return to my pharmacy for future care**. |  |  |  |  | |  |  |
| 15. Screening for TB symptoms and referring eligible people for chest X-ray will help to **identify** **more people with TB**. |  |  |  |  | |  |  |
| 16. Screening for TB symptoms and referring eligible people for chest X-ray will help **increase patient trust in my pharmacy.** |  |  |  |  | |  |  |
| 17. I feel **confident in my ability** **to screen** my customers for TB symptoms using the ACIS form in the SwipeRx app. |  |  |  |  | |  |  |
| 18. I feel **confident in my ability to** **refer** eligible people for chest X-ray. |  |  |  |  | |  |  |
| 19. I believe that TB symptom screening and chest X-ray referrals would be **beneficial to my pharmacy.** |  |  |  |  | |  |  |
| 20. I believe that TB symptom screening and chest X-ray referrals would **differentiate the service provided by my pharmacy** from other pharmacies in the area. |  |  |  |  | |  |  |
| 21. I am worried that the screening app would **not be able** to keep my **customers’ information confidential**. |  |  |  |  | |  |  |
| 22. The **incentives motivated** me to participate in the project. |  |  |  |  | |  |  |
| 23. I feel the incentives would compensate me fairly for the time and effort **required to participate** in the project. |  |  |  |  | |  |  |

The last 2 questions of the survey use a different scale ranging from Very Difficult to Very Easy. Please respond below:

| **Questions** | **Very difficult** | **Difficult** | **Neither difficult nor easy** | **Easy** | **Very Easy** | **Refuse/**  **Unknown** |
| --- | --- | --- | --- | --- | --- | --- |
| 24. My customers would perceive **the time required to screen** them on the ACIS form in the SwipeRx app as… |  |  |  |  |  |  |
| 25. My customers would perceive the **time required to get a chest X-ray** and be evaluated for TB as… |  |  |  |  |  |  |

26. If you could make any one change to the project, what would you change?

27. If you haven’t made any referral for the apps, what was the reason you did not make referrals?
