# Supplementary material for: Evaluating novel engagement mechanisms, yields and acceptability of tuberculosis screening at retail pharmacies in Ho Chi Minh City, Viet Nam
Source: PLOS Glob Public Health. 2022 Oct 17;2(10):e0000257. doi: 10.1371/journal.pgph.0000257 (PMC10021543; doi:10.1371/journal.pgph.0000257)
Supplement: S2 Survey — (DOCX) [file pgph.0000257.s007.docx]

| **Trước khi khảo sát** |  |
| --- | --- |
| **Mã số ACIS***:* **____________________** | **Mã số SwipeRx: ______________________** |
| **Dược sĩ có thực hiện chuyển gửi chưa?** [ ] Có [ ] Không | |
| **Câu hỏi cần trả lời** |  |
| **1. Ngày phỏng vấn***:* *__ __/ __ __/__ __ __ __* | **2. Tên người phỏng vấn:** *__________________* |
| **3. Năm sinh:** *__ __ __ __* | **4. Tên dược sĩ:**  *__________________* |
| **5. Số năm làm việc tại nhà thuốc tư***: __________năm* | |
| **6. Năm tốt nghiệp trường Dược?***: __ __ __ __* | |

**Hướng dẫn**: Xin vui lòng đọc những câu dưới đây cho dược sĩ và yêu cầu họ chọn một câu trả lời nằm trong thang điểm từ Rất Đồng ý đến Rất Không đồng ý. Trong những câu dưới đây, “anh/chị” có nghĩa là dược sĩ.

| **Câu hỏi** | **Rất đồng ý** | **Đồng ý** | **Bình thường** | **Không đồng ý** | | **Rất không đồng ý** | **Từ chối/**  **Không biết** |
| --- | --- | --- | --- | --- | --- | --- | --- |
| **7. Tập huấn** anh/chị nhận được từ nhân viên dự án là đầy đủ để anh/chị có thể sử dụng mẫu ACIS trên SwipeRx app. |  |  |  |  | |  |  |
| 8. Nếu khách hàng có dấu hiệu và triệu chứng bệnh lao, việc dược sĩ tầm soát lao bằng bản câu hỏi **là phù hợp**. |  |  |  |  | |  |  |
| 9. Nếu khách hàng có dấu hiệu và triệu chứng lao, việc dược sĩ chuyển gửi bệnh nhân làm **các xét nghiệm chẩn đoán** không cần tư vấn của bác sĩ là **hợp lí.** |  |  |  |  | |  |  |
| 10. Bằng việc sử dụng mẫu ACIS trong SwipeRx app để tầm soát triệu chứng lao và chuyển gửi đi chụp Xquang phổi, anh/chị có thể giúp khách hàng của mình được **chẩn đoán ra lao sớm hơn**. |  |  |  |  | |  |  |
| 11. Bằng việc sử dụng mẫu ACIS trong SwipeRx app để tầm soát triệu chứng lao và chuyển gửi đi chụp Xquang phổi, anh/chị có thể đảm bảo những khách hàng mắc lao nhận được **các xét nghiệm chẩn đoán đảm bảo chất lượng**. |  |  |  |  | |  |  |
| 12. Việc tầm soát các triệu chứng bệnh lao và chuyển gửi những người đủ tiêu chuẩn đi chụp Xquang phổi lấy mất **quá nhiều thời gian có thể làm những việc khác** của anh/chị**.** |  |  |  |  | |  |  |
| 12a. Nếu câu trả lời= Đồng ý hay Rất Đồng ý=> Nếu phiếu thu thập dữ liệu ngắn hơn, anh/chị có còn cảm thấy việc tầm soát lấy mất quá nhiều thời gian cho các hoạt động khác? | **[ ] Có** | | **[ ] Không** | | **[ ] Từ chối/Không biết** | | |
| 13. Việc tầm soát các triệu chứng bệnh lao và chuyển gửi những người đủ tiêu chuẩn đi chụp Xquang phổi có thể **làm anh/chị** **bị mất chi phí**, có thể là chi phí gián tiếp hay chi phí trực tiếp**.** |  | |  | |  | | |
| 14. Anh/chị lo rằng việc tầm soát các triệu chứng lao và chuyển gửi những người đủ tiêu chuẩn đi chụp Xquang phổi có thể **khiến cho khách hàng của anh/chị không quay lại nhà thuốc để mua thuốc lần sau**. |  | |  | |  | | |
|  |  | |  | |  | | |
| **Câu hỏi** | **Rất đồng ý** | **Đồng ý** | **Bình thường** | **Không đồng ý** | | **Rất không đồng ý** | **Từ chối/**  **Không biết** |
| 15. Việc tầm soát triệu chứng lao và chuyển gửi những người đủ tiêu chuẩn đi chụp Xquang phổi **sẽ giúp phát hiện nhiều bệnh nhân lao**. |  |  |  |  | |  |  |
| 16. Việc tầm soát triệu chứng lao và chuyển gửi những người đủ tiêu chuẩn đi chụp Xquang phổi sẽ làm **tăng sự tin tưởng của bệnh nhân vào nhà thuốc của anh/chị**. |  |  |  |  | |  |  |
| 17. Anh/chị **tự tin mình có khả năng tầm soát triệu chứng lao** cho khách hàng của mình bằng việc sử dụng mẫu ACIS trên SwipeRx app. |  |  |  |  | |  |  |
| 18. Anh/chị **tự tin vào khả năng chuyển gửi** **của mình** cho những người đủ tiêu chuẩn đi chụp Xquang phổi. |  |  |  |  | |  |  |
| 19. Anh/chị tin là việc tầm soát triệu chứng lao và chuyển gửi cho Xquang phổi **sẽ có lợi cho nhà thuốc của anh/chị**. |  |  |  |  | |  |  |
| 20. Anh/chị tin việc tầm soát triệu chứng lao và chuyển gửi đi chụp lao phổi **sẽ giúp cho dịch vụ của nhà thuốc anh/chị** **khác biệt** so với các nhà thuốc khác trong khu vực. |  |  |  |  | |  |  |
| 21. Anh/chị lo lắng rằng ứng dụng tầm soát **sẽ không thể** giữ **bí mật** **thông tin khách hàng của anh/chị**. |  |  |  |  | |  |  |
| 22. Khoản tiền **bồi dưỡng đã khuyến khích** anh/chị tham gia dự án. |  |  |  |  | |  |  |
| 23. Anh/chị cảm thấy **khoản tiền bồi dưỡng** đền bù **xứng đáng** cho thời gian và công sức anh/chị **dành để tham gia** dự án. |  |  |  |  | |  |  |

2 câu hỏi cuối cùng sử dụng một thang điểm khác từ Rất Khó đến Rất Dễ. Xin trả lời câu hỏi này:

| **Câu hỏi** | **Rất khó** | **Khó** | **Không khó không dễ** | **Dễ** | **Rất dễ** | **Từ chối/Không biết** |
| --- | --- | --- | --- | --- | --- | --- |
| 24. Khách hàng của anh/chị có thể nghĩ rằng **thời gian yêu cầu cho tầm soát** trên mẫu ACIS trên SwipeRx app là… |  |  |  |  |  |  |
| 25. Khách hàng của anh/chị có thể nghĩ rằng **thời gian yêu cầu cho việc đi chụp Xquang phổi** và để đánh giá bệnh lao là… |  |  |  |  |  |  |

26. Nếu anh/chị có thể thay đổi một điều ở dự án, anh/chị sẽ thay đổi gì?

27. Nếu anh/chị chưa từng chuyển gửi cho dự án, tại sao anh/chị không chuyển gửi?
