# Supplementary material for: Evaluating novel engagement mechanisms, yields and acceptability of tuberculosis screening at retail pharmacies in Ho Chi Minh City, Viet Nam
Source: PLOS Glob Public Health. 2022 Oct 17;2(10):e0000257. doi: 10.1371/journal.pgph.0000257 (PMC10021543; doi:10.1371/journal.pgph.0000257)
Supplement: Attachment — Submitted filename: TDR Manuscript - Response to Reviewers.docx [file pgph.0000257.s009.docx]

**Response to Reviewers**

**Additional Editor Comments:**

Abstract:

Line 27: Recommend clearly linking in the second or third sentence that SwipeRx platform is a professional networking app. As written, it is implied but not fully clear.

We modified the text starting on Line 28 “Between September and December 2019, we piloted the use of a social networking app, SwipeRx, to recruit pharmacists for a TB referral scheme across four districts of Ho Chi Minh City, Viet Nam.”

Line 37: Add a comparator for the sentence below to better contextualize results (e.g., compared to whom?).

We have rewritten the sentences starting on Line 37 as suggested: “Compared to pharmacists who did not make any CXR referrals, pharmacists making at least one CXR referral understood the pilot’s objectives more clearly (aOR=2.6, 95% CI: 1.2-5.8) and believed that TB screening increased customer trust (aOR=2.7, 95% CI: 1.2-5.8), benefited their business (aOR=2.8, 95% CI: 1.3-6.2) and constituted a competitive advantage (aOR=4.4, 95% CI: 1.9-9.9). They were also more confident in using mHealth apps (aOR=3.1, 95 CI%: 1.4-6.8).”

Line 43: For the sentence, as is written, “Digital networking platforms, such as SwipeRx, can facilitate pharmacist engagement, but…” does not seem to be supported by the results provided (e.g., at best 78/1816 ~4.3%; or 1/1816 within the intervention area ~0.6%). I would suggest that you more clearly demonstrate what supports this statement (e.g., that referrals were made through app after in-person outreach), or provide greater nuance to your conclusion in the abstract.

We have rewritten the sentence starting on Line 43 as suggested: “Digital networking platforms, such as SwipeRx, can facilitate referrals for TB screening by pharmacists, but their ability to identify and recruit pharmacists requires optimization, particularly when targeting specific segments of a nation-wide digital network.”

Manuscript:

Methods: For the acceptability survey, how did the author’s decide which sub-set from each group to recruit (e.g., the 50/54 and the 56/92)? Please provide clarification how you decided whom to survey from among those that participated.

The acceptability section of the methods has been restructured and rewritten. Phamacists in each survey cohort were randomly approached for recruitment. The sentence start on Line 147 has been updated to better highlight this recruitment approach: “Pharmacists who made at least one CXR referral which resulted in the participant getting a CXR screen via the pilot (n=50), as well as pharmacists who agreed to participate, but did not make any CXR referrals (n=50), were randomly approached for recruitment until the sample size requirements were met.”

Line 139: Please reference S2 Table after noting checking internal consistency via Cronbach’s alpha.

Text referencing the S2 Table has been inserted in Line 142.

Table 1: Recommend somehow capturing the two different methods of outreach. As is, it’s a bit misleading since most people outreached via a push notification declined to take part.

The strategy of engaging pharmacies to participate in the pilot via the SwipeRx app did not result in any recruitment. Only one willing pharmacist was identified and when she was contacted, she actually did not work in the pilot’s intervention distircts and thus was ineligible. The data in Table 1 refer only to in-person recruitment efforts by the pilot staff. We introduced a paragraph break between Lines 183 (SwipeRx engagement) and 184 (in-person engagement), and modified the title of Table 1 to more clearly highlight these data only cover the in-person recruitment efforts.

Line 204: The results text here mentions 6 acceptability factors across four constructs, yet the methods text mentions 7 acceptability constructs. Please reconcile these differences. Do the authors mean that 4 of 7 acceptability constructs were higher among those making at least one referral?

Our survey assessed perceptions of seven acceptability constructs across multiple prompts (factors). The text starting in Line 217 has been rewritten in like with the reviewer’s suggestions: “There were significant differences in pharmacist responses for six factors associated with four of the seven acceptability constructs (Table 4).”

Discussion/limitations:

Given the very low engagement following digital outreach, were there any efforts to understand the characteristics of pharmacists invited via push notification but who did not participate (and how they compared to those who agreed) and their barriers to engagement / reasons for non-participation? Since this constituted 95% of those originally outreached to, it would be extremely informative to understand determinants related to why they didn’t accept/participate. If this data was not collected, please note this as a study limitation.

We did not collect information on pharmacists who invited to participate via the push notification, but did not elect to participate. We have added this as a study limitation in text starting on Line 308: “Another limitation is that we did not collect any information about the characteristics and barriers faced by pharmacists who did not respond to the push notifications encouraging them to participate in the pilot via the SwipeRx app.”

Conclusion:

Please provide greater nuance to your conclusions as currently the challenges of engagement/adoption around digital outreach are not mentioned. This is an important aspect of your findings (e.g., while adoption via digital outreach was low, among those reached, the screening intervention was highly acceptable).

The conclusion text (Lines 319-326) has been rewritten to better highlight the challenges with the pilots digital outreach approach. “The engagement of pharmacists for TB screening and referrals shows promise in this urban, high TB burden setting, as a high yield of people with TB have the potential to be identified earlier in their disease course and screening and diagnostic referrals were highly acceptable among participating pharmacists. In order to efficiently engage pharmacies for this activity at scale, more evidence on the optimal use of online and offline channels of recruitment is needed, particularly the role of professional networking and social media in identifying and reaching eligible collaborators. Despite these unanswered questions, this pilot adds to the evidence base regarding the inclusion of pharmacies in a country’s private sector engagement strategy and their strategies to end TB overall.”

**Reviewer 1**

**Major comments**

My major comments are around the reporting of the acceptability survey. The Introduction states that the intention was to identify factors with a pharmacist’s active participation in the pilot, and the Methods state that recorded referral was the primary outcome and attitudes the primary exposure. However, in the results sentences are phrased as if the modelled association was the other way around – for example “those who referred were also more likely to agree that the pilot would increase customer trust…”. I have several related comments here:

It’s not completely clear to me from description in methods and results including Table 3 what way around the multivariable model is set up. I think that, as currently reported, these are probably odds ratios for a one unit increase on a five-item Likert scale to predict whether a pharmacist has made a referral or not? i.e. the left hand side of model is whether a referral was made or not, and the right hand side is the attitude score (p[made a referral] ~ [attitude score] + [other covariates] + error). I would be grateful if the authors could provide additional details to clarify what exactly is being modelled here, and which model structures were used.

However, I think because of the way the pharmacists have been purposefully sampled that the exposure (i.e. attitudes) needs to be on the left hand side of the model and whether someone made a referral or not on the right hand size. Looking at the methods, it seems as if pharmacists were purposefully sampled to separately recruit equal numbers of those who did and did not refer patients. I think this is probably equivalent to a case-control study. It follows therefore that the authors should be aiming to compare exposures (i.e. acceptability measures) between pharmacists with given levels of outcome. I don’t think they can directly estimate odds ratios for outcomes (referral vs. no referral) since data were collected / pharmacists were purposefully sampled conditional on whether a pharmacist had made a referral or not, and therefore the authors already determined that half of all included pharmacists had made a referral. Unless there is something I have missed, I think the correct model set-up would be [attitude scale measure] ~ p[made a referral] + [other co-variates] + error. Because attitude measures are on a 5-level scale, I think this would probably best be a multinomial model. A multinomial model would avoid making assumptions of linearity (or log-linearity) between the Likert scale levels (i.e. would avoid assumption that a difference from 1 to 2, it the same as the difference from 3 to 4). The estimand here then wouldn’t be an odd ratios, but an expected difference in attitude score in those who did vs. those who did not make a referral.

We have made extensive changes to the acceptability survey data analysis and manuscript Methods and Results sections in line with the reviewer’s feedback. Table 4 was updated to show the results of mixed-effect ordinal proportional odds models to explore the association of individual factors as the primary outcome and a recorded referral as primary exposures.

Although it is mentioned by the authors, I wonder if there was much evidence for clustering of attitudes by District? If so, I don’t think these results (i.e. district-specify effects and variation) have been fully reported. I wonder if the authors might increase the precision of estimates if they considered responses nested within pharmacists rather than districts (for example, the pharmacists who think screening takes too much time are also likely to think screening costs pharmacists too much money). I think this would require modelling of all attitude scores at once, rather than in many separate models, but I think this could probably be handled in an item-response model. This is just a suggestion.

We conducted an additional analysis to show differences in acceptability constructs across the four pilot districts. Since the differences were limited, the manuscript text starting in Line 224 was updated acknowledging these differences and data are presented in the S4 Table. We did not construct an item-response model; this survey’s analysis follows a similar approach and presentation as other publicaitons which use the same acceptability framework.

In Table 3, rather than showing mean and median attitude response, the authors should show numbers and proportion of people selecting each 1 to 5 responses. If it can’t sensibly fit into a table, I think it should at least be supplementary material.

The manuscript text on Line 227 was updated indicating that a detailed description of survey responses is provided in the S5 Table.

Understanding of this would be clearer if an equation was included in the manuscript, or the statistical code was made available (I note that PLoS Global Public Health recommends that code be made freely available).

The equation for the final model is now included in the Methods section in the text starting on Line 160. Stata code is also provided in the supplementary materials.

For the sample size calculation, the authors write that the sample size is based on the “probability of a pharmacist making referrals when their attitude is at the mean of a five point likert scale”. But when the probability of referral is what? What probability of referral at the mean of five point scale did they use? That said, I think if the analysis substantially changes this power calculation may become moot.

The text referring to the survey’s sample size was updated starting in Line 143: “We calculated a sample size of 100 pharmacists, in two equal sized cohorts of 50, based on a 2-sample, 2-sided comparison of mean scores on a 5-point Likert scale, with a 95% confidence level and 80% power. We estimated an average rating difference between pharmacists of 0.9 and a population variance of 5 based on initial results from piloting the survey instrument.”

**Minor comments**

I would be interested to read a little more in introduction or methods about the healthcare system set-up in Viet Nam. For instance, the paper mainly talks about “pharmacists” but sometimes about “pharmacy professionals”. Are all the people included qualified pharmacists? (vs. people who work in retail pharmacy, perhaps under supervision of an off-site pharmacist)? For instance, in Malawi, if I went to pharmacy retail outlet, the person I would speak with would be unlikely to be a trained pharmacist, although a trained pharmacist might be responsible for the retail outlet overall. What is the regulatory framework in Viet Nam? What level of medical education do these people tend to have? Similarly, in comments about private vs. public sector I’d be interested to know about costs in the public sector, are chest Xrays free? And what are costs to people for TB treatment in public vs. private sector? This need not be long, but a sentence or two would help me understand a little more about the system in which the intervention is situated.

The manuscript has been updated throughout to use a consisten naming scheme for pharmacists. The background section has been updated starting on Line 60 to highlight how pharmacies operate in Viet Nam: “Most retail pharmacies in Viet Nam employ staff with minimal formal pharmacy training, who then work under the supervision of an off-site pharmacist.” In addition, the text start on Line 112 has been updated to indicate that the pilot engaged with anyone working the pharmacy “directly dispensed and consulted clients in the pilot, regardless of their official certification or degree.” The text starting on Line 123 highlights the cost which participants paid for CXR: “The actual cost of a CXR at participating radiology sites ranged from VND 80,000 (USD 3.46) to VND 120,000 (USD 5.19), with the presumptive TB patient paying the difference [after the VND 50,000 (USD 2.16) subsidy was applied].” The cost for a CXR in public sector is within this range and varies depending on health facilities. We did not modify the text to discuss the cost of public vs private TB care, as we felt this is outside the scope of the screening and diagnostic referral pilot.

In some places the authors talk about “made a referral” vs. “made a successful referral”. What is the definition of making a “successful” referral? Does this require the referred person to actually receive a chest Xray? Or just that it went through on the mHealth app?

A successful CXR referral is one where the participant eventually got screened by CXR via our pilot. We have modified the text throughout the manuscript to remove the word successful.

In line 114 to 122, the authors talk about people with “suspected TB”. Whilst “people with suspected TB” is infinitely better than “TB suspects”, I still think this isn’t preferred language. Would “people with presumptive TB” or “people who had been identified as having TB symptoms” be better?

We modified the text of the manuscript so that presumptive TB is used throughout.

In line 252 the authors write about what the pharmacists think their customers want from a service and line 277 about how participating in the pilot might lead to increased trust from customers. I would perhaps add to the discussion the limitation that only pharmacists were surveyed, so we don’t directly know what pharmacy customers perceive / prefer or what would actually lead to an increase in customer trust - merely what the pharmacists themselves think.

We added text starting on Line 300 highlighting the need to assess the perceptions of pharmacy clients and pilot beneficiaries: “Further studies assessing the perspective of pharmacy customers and beneficiaries of the diagnostic referral pilot are also needed, to understand whether the pilot and its promotion of champion referrers would actually result in increased trust in the champion pharmacy.”

The authors probably don’t need both p values and confidence intervals, confidence intervals are probably sufficient on their own.

We removed p-values from the manuscript text and keep only confidence intervals.

**Trivial comments or typos**

I am not sure “bifurcated” is the most usual word to use for dividing data. Would “divided according to” be better?

We have changed the word “bifurcated” to “divided”.

The authors refer throughout to “multivariate” models, I think they probably mean “multivariable” (although if they change the modelling approach as suggested above, this might change).

We revised the Methods section in line with comments above, which resulted in a change of model type.

Line 233 to 235 seems overly long, could this be split up? Something along the lines of, “Probably pharmacists conducted ‘pre-screening’ of customers to only refer those with more severe symptoms rather than all those meeting symptom screening criteria, likely depressing the total number of referrals. Overall the intervention resulted in detection and linkage to care of seven persons with TB among 182 persons referred for CxR screening”?

We split this sentence into two as suggested.

**Reviewer 2**

It would be good to have a bit more information about pharmacies in Viet Nam and the pharmacists included in the study ie. do the pharmacists tend to work alone in their pharmacy or are they working alongside others? Is it the trained pharmacist who might be working in the background while an assistant is the main interface with customers?

The background section has been updated starting on Line 60 to highlight how pharmacies operate in Viet Nam: “Most retail pharmacies in Viet Nam employ staff with minimal formal pharmacy training, who then work under the supervision of an off-site pharmacist.” In addition, the text start on Line 112 has been updated to indicate that the pilot engaged with anyone working the pharmacy “directly dispensed and consulted clients in the pilot, regardless of their official certification or degree.”

Line 87: How were your pilot districts selected?

Text has been added describing how districts were selected, starting in Line 91: “These districts were selected so that the pilot was embedded within the larger PCPS project, which at the time focused on engaging doctors and hospitals for TB diagnostic referrals.” These districts are part of the dense urban core of Ho Chi Minh City, primarily located near Pham Ngoc Thach Hospital, where the Provincial TB Program is located.

Lines 97 & 98: What is the definition of eligibility? Were push notifications only sent to pharmacists in the pilot area? Later in the results it appears this was not the case but it would be good to have this clearer in the methods

We modified the text started in on Line 102 to highlight how push notificaitons were sent: “We sent push notifications through the SwipeRx app to active user accounts registered in Ho Chi Minh City a total of five times over a one-month period; at the time, further targeting at the district-level was not possible. We also launched a Facebook campaign encouraging pharmacists in our intervention districts to join SwipeRx and to participate in the pilot.”

Line 124: What is meant by “successful CRX”. Is this that only referral was made, or referral and CXR done, or that the patient was TB positive? Suggest removing the word “successful”.

A successful CXR referral is one where the participant eventually got screened by CXR via our pilot. We have modified the text throughout the manuscript to remove the word successful.

Line 133: Is there a reference for theoretical framework of acceptability?

We cited reference #26 so readers can learn more about the Sekhon et al. acceptability framework.

Line 172: I would argue that your 84.4% who installed the SwipeRx rather overstates participation. This should instead be 146 of the 253 approached (58%).

We agree that ultimately 57.7% of those approached (146/253) installed the SwipeRx app. The 84.4% proportion uses pharmacists ‘agreeing to participate’ as the denominator, and was included in the text so that the proportion matched the one presented in the cascade of recruitment in Table 1.

Lines 173-175: Its seems that 207 notifications were sent to the 146 participating pharmacists which is just over 1 notification per pharmacist over the 4-month period. This doesn’t seem very much given the supposed ease of doing these notifications. Can you please clarify and/or discuss this more in the discussion.

The text starting on Line 186 was rewritten. The reminder messages were only sent during a 2-month period in the second half of the pilot. Each reminder contained different messages and was sent to a quarter of the people who were collaborating with the pilot at that moment. We had hoped to test whether certain messages resulted in more active participation, but opening of the push notifications and resulting changes in referral behaviour was minimal.

Line 180 & 181: 54 pharmacists referred patients for CXR: 2 pharmacists made many referrals and 35 made just one or two. What happened to the other 27 pharmacists?

The text starting on Line 195 has been updated to show that the remaining 17 pharmacists made between three to five referrals each.

Lines 190-193: How did you recruit the pharmacists who made zero referrals? Was this random selection?

The acceptability survey section of the Methods was restructured and rewritten. The text start on Line 147 now more clearly indicates that pharmacists were randomly approached. This is also reflected in the results section in the text starting on Line 203.

Line 244: Pharmacist feedback - “unwillingness to give false advice”. I didn’t see any reference to this in the results. Was this informal feedback or was it part of the questions in Table 4?

The text starting on Line 256 has been rewritten to relfect that this insight originates from work done by Lönnroth et al: “A pharmacist’s lack of confidence in making referrals could be driven by an unwillingness to give false advice, particularly about a stigmatized disease, such as TB.”

11. Lines 296-298: Only 35% of patients referred actually received a CXR. This seems a very small proportion yet you have left it to just these two lines to mention it. I realise your paper is more about referral but it would be good to see more discussion on this.

The paragraph starting on Line 265 has been modified to more explicity discuss challengs with the low rate of CXR screening after referral in line with the reviewr suggstions.
