# Supplementary material for: Evaluating novel engagement mechanisms, yields and acceptability of tuberculosis screening at retail pharmacies in Ho Chi Minh City, Viet Nam
Source: PLOS Glob Public Health. 2022 Oct 17;2(10):e0000257. doi: 10.1371/journal.pgph.0000257 (PMC10021543; doi:10.1371/journal.pgph.0000257)
Supplement: Attachment — Submitted filename: Rebuttal Letter 2022-08-25.docx [file pgph.0000257.s010.docx]

**Rebuttal Letter**

**Journal Requirements:**

1. Please update your online Competing Interests statement. If you have no competing interests to declare, please state: “The authors have declared that no competing interests exist.”

This was updated in the online portal.

2. Please review your reference list to ensure that it is complete and correct. If you have cited papers that have been retracted, please include the rationale for doing so in the manuscript text, or remove these references and replace them with relevant current references. Any changes to the reference list should be mentioned in the rebuttal letter that accompanies your revised manuscript. If you need to cite a retracted article, indicate the article’s retracted status in the References list and also include a citation and full reference for the retraction notice.

We reviewed the reference list and noticed some formatting errors. These have been corrected in track changes. In addition, one citation was dropped as it was used in text about a map which was dropped from an earlier version of the manuscript.

**Additional Editor Comments:**

- Abstract: Kindly structure your abstract according to journal’s formatting guidelines.

The abstract was updated so that it is presented in three sections, in line with the online submission guidelines for authors.

- Please ensure that the remainder of your manuscript accords with the journal’s formatting guidelines.

We reviewed the guidelines to ensure the manuscript complies with formatting guidelines.

- Please carefully review the text of your manuscript one final time for any small grammatical errors or typos as there will not be a formal proofing stage, nor a further opportunity to revise your manuscript.

We carefully reviewed the manuscript and made several small changes to the text to fix spacing and grammatical error issues.

**Reviewers' Comments:**

On Page 3 you say “Most retail pharmacies in Viet Nam employ staff with minimal formal pharmacy training, who then work under the supervision of an off-site pharmacist”. Then on Page 5 you say “We engaged any staff member working at the pharmacy who directly dispensed and consulted clients in the pilot, regardless of their official certification or degree”. Did your definition of pharmacist and hence the recruitment include only the “off-site pharmacist” or did it also include those with no official training? It seems that it is the latter but then Table 3 makes it seem like it was only trained pharmacists. It would be good to clarify this and also the implications of this in the discussion.

Thank you for this comment. We did engage any staff member working at a pharmacy who was directly interacting with clients, in line with the description on Page 3. We have added text to indicate that throughout the remainder of the manuscript, we refer to these individuals as pharmacists, even if they are not qualified or officially licenced. In addition, the results text on Page 5 has been modified to indicate we approached staff working in pharmacies – not just qualified and licensed pharmacists.
